# A systematic review of outcomes associated with patients admitted to hospital with emergency haematuria

**DOI:** 10.1002/bco2.497

**Published:** 2025-02-17

**Authors:** Nikki Kerdegari, Raghav Varma, Simona Ippoliti, Cameron Alexander, Arjun Nathan, Kevin Gallagher, Sinan Khadhouri, Kevin Byrnes, Nikita Bhatt, Veeru Kasivisvanathan

**Affiliations:** ^1^ GKT School of Medical Education King's College London London UK; ^2^ Department of Urology Sandwell and West Birmingham Hospitals NHS Trust UK; ^3^ Department of Urology Hull University Teaching Hospitals Hull UK; ^4^ Department of Urology Colchester General Hospital Colchester UK; ^5^ Department of Urology Whipps Cross Hospital London UK; ^6^ Department of Urology Western General Hospital Edinburgh UK; ^7^ School of Medicine University of St Andrews St Andrews UK; ^8^ Department of Urology North Middlesex University Hospital London UK; ^9^ Department of Urology Newcastle upon Tyne Hospitals NHS Foundation Trust Newcastle‐upon‐Tyne UK; ^10^ Division of Surgery and Interventional Science University College London London UK

**Keywords:** bladder cancer, emergency, unscheduled haematuria

## Abstract

**Objective:**

Unscheduled admission for haematuria accounts for 15% of all urological emergencies with over 25 000 patients admitted each year in the UK. It is associated with prolonged admission and poor clinical outcomes. This systematic review aims to determine current management strategies and outcomes in these patients.

**Methods:**

A systematic search was performed in October 2023 across MEDLINE, EMBASE and Web of Science for randomised controlled trials and retrospective and prospective observational studies assessing the management of patients admitted as an emergency with haematuria. The primary outcome measure was the length of stay (LoS). Secondary outcomes included hospital readmission, mortality and health resource use.

**Results:**

Three eligible publications with a total of 219 patients were identified. Mean length of stay was 5.8 days. The pooled mean age of unscheduled emergency haematuria was 74.8 years and 87.9% of patients were male. Bladder cancer was present in 17% of patients and, similarly, prostate cancer was present in 17% of patients.

**Conclusions:**

Unscheduled admission for haematuria is associated with long LoS. This systematic review has demonstrated a lack of data reporting outcomes of unscheduled haematuria and its management strategies. There is a need to perform large‐scale prospective studies to better understand this cohort of patients.

## 1 INTRODUCTION

Unscheduled admissions to hospitals with visible haematuria accounts for 15% of clinical diagnoses of urological emergencies in the United Kingdom with over 25 000 patients admitted each year.^1^

The management of this condition is often complicated by comorbidities and the use of antiplatelet or anticoagulant drugs.^2^, ^3^ In a retrospective series, this population of patients had an average ASA score of three and mean age 78 years, with a 30‐day mortality rate of 5% and a one‐year all‐cause mortality rate of 23%.^4^

There are no recognised standardised protocols or guidelines for managing haematuria as an emergency. Inpatients admitted with haematuria often have long inpatient stays and are often readmitted with the same presentation. The median UK inpatient stay for haematuria patients has increased from 8.5 to 10 days over the last five years, contrary to the drive towards shorter stays in elective urological surgeries.^5^, ^6^ In addition to these poor clinical outcomes, patients require high resource use. A recent study examining resource use associated with radiation cystitis in a single centre estimated a cost burden of €23 706 per patient. Across 56 patients, 621 inpatient bed days were used, averaging 11 days per patient.^7^ Of note, in the IDENTIFY study cohort (an observational study of the investigation of haematuria in secondary care), 32% of those admitted as an emergency with haematuria had an underlying malignant diagnosis.^8^ This has important implications for prompt identification and management pathways for these underlying cancers.

There are no clear evidence‐based guidelines dictating the timing of inpatient investigation and definitive treatment for these patients having failed conservative management. Consequently, the management of unscheduled haematuria is likely to vary both nationally and internationally. The aim of this systematic review is to determine the effectiveness of current management strategies in the management of patients with unscheduled, emergency haematuria hospital admissions.

## 2 METHODS

### 2.1 Search strategy

This systematic review was conducted in accordance with the Preferred Reporting Items for Systematic Review and Meta‐Analysis (PRISMA) guidelines.^9^ The study protocol was registered on PROSPERO (CRD42023466710). A structured literature search of the MEDLINE, Embase and Web of Science databases was performed for studies conducted between January 2013 and October 2023 to capture contemporary, modern clinical practice. The full search strategy is provided in Supplementary material.

### 2.2 Study selection, inclusion and exclusion criteria

Randomised controlled trials (RCTs) and observational studies on the management of patients admitted with unscheduled haematuria were included. Only articles in English, on patients 16 years of age or older and admitted with unscheduled haematuria as the primary or secondary diagnosis were included. Review articles, case studies and conference abstracts were excluded. The data was pooled for studies reporting the primary outcome. Other articles that did not meet the primary outcome but included relevant secondary outcomes were also included and reported separately.

Three authors (NK, RV and SI) were involved in independently screening abstracts and full‐text articles. Each abstract and full‐text article was screened by two authors. Where agreement was not reached between the two authors, the third author acted as an arbiter.

### 2.3 Data extraction

Data from studies that met the inclusion criteria was independently analysed by two reviewers (NK and RV) and input into a standardised case report form. Data was collected on study characteristics such as author, year of publication, study type and number of patients included. Patient‐related factors collected included age, gender, concomitant comorbidities, previous urological history and clinical presentation of haematuria. Disease‐related factors included underlying aetiology, number of previous admissions for the same reason, findings on cystoscopy or other investigations, level of care required. Intervention‐related data extracted included the specific investigation and therapeutic measures implemented to manage patients. Outcome data collected included length of stay (LoS), 30‐day and 90‐day readmission rates, 30‐day and 90‐day mortality rates, transfusion rate, rate of thromboembolic events and complications associated with the procedures performed. The primary outcome measure was LoS. Secondary outcomes included incidence, presentation, identification of underlying aetiology, hospital readmission, management strategies, mortality and health economic evaluation. Where homogeneity of data was present, pooled estimates were obtained using means for continuous variables while event rates were obtained for dichotomous variables.

### 2.4 Statistical analysis

A meta‐analysis was conducted if more than one randomised (or quasi‐randomised) controlled trial (RCT) reported the same outcome. For studies with multiple publications, only the most recent or complete data were used. Non‐randomised studies were not included in the quantitative analysis. A fixed effects model was applied for pooled treatment effect estimates unless heterogeneity was expected, in which case a random effects model was used.

For time‐to‐event data, hazard ratios were combined using the inverse variance method. Dichotomous outcomes were combined using the Mantel–Haenszel method, while continuous outcomes used the inverse variance method. If different scales were used, standardised mean differences were calculated. If meta‐analysis was not feasible, results were summarised narratively.

### 2.5 Risk‐of‐bias assessment

Risk of bias assessment was undertaken independently by two authors (NK and RV). The Risk Of Bias In Non‐randomised Studies ‐ of Interventions (ROBINS‐I) tool was used for non‐randomised studies. The Risk of Bias (RoB2) revised Cochrane tool was used for RCTs. Any disagreements were resolved by discussion.

## 3 RESULTS

### 3.1 Study characteristics

**FIGURE 1:**
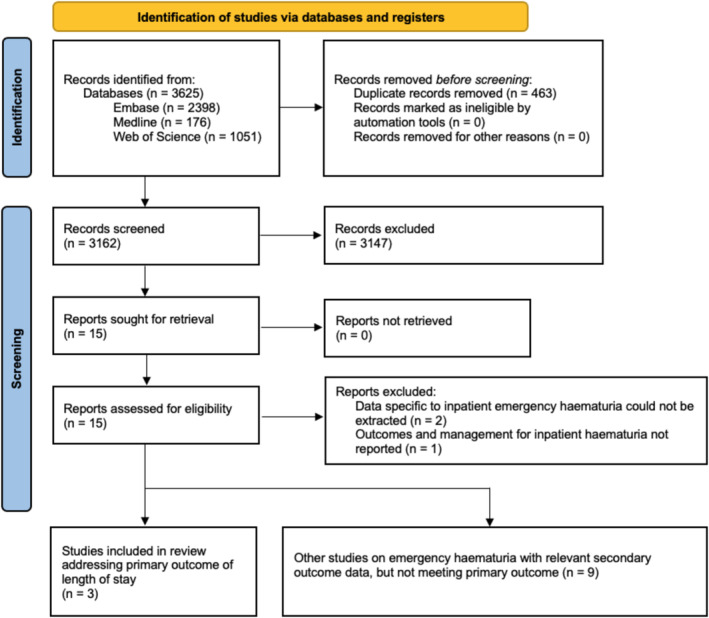
PRISMA flowchart of studies. *From:* Page MJ, McKenzie JE, Bossuyt PM, Boutron I, Hoffmann TC, Mulrow CD, et al. The PRISMA 2020 statement: an updated guideline for reporting systematic reviews. BMJ 2021;372:n71. doi: 10.1136/bmj.n71 For more information, visit: http://www.prisma‐statement.org/

Figure 1 describes the PRISMA flowchart of study selection. Fifteen articles were retrieved for full‐text screening of which three retrospective cohort studies reported the primary outcome and were included in the final review. Three articles were excluded due to either the inability to extract data specific to inpatient unscheduled haematuria or outcomes and management for inpatient haematuria were not reported. The remaining nine articles did not report the primary outcome but included secondary outcomes on inpatient unscheduled haematuria management which were separately tabulated.

Due to the lack of sufficient RCTs in the systematic review, it was not possible to perform a meta‐analysis. A narrative synthesis of the results was hence performed.

### 3.2 Patient characteristics

Baseline characteristics of the three included studies, with a total of 219 patients, are shown in Table 1. Two retrospective cohort studies^4^, ^10^ with a combined total of 198 patients revealed a pooled mean age of 75 years, 88% of male patients and 45% patients on antithrombotic therapy. Another retrospective cohort of 21 patients with radiation cystitis^11^ reported a median age of 73 years, 91% male and 27% on antithrombotic therapy.

**TABLE 1:** Baseline data of articles meeting eligibility criteria for inclusion in the systematic review.

	Sönmez et al.^10^	Pavithran et al.^4^	Wallace et al.^11^
**Study type**	Retrospective cohort	Retrospective cohort	Retrospective cohort
**Year**	2023	2022	2022
**Total number of patients**	78	120	21
**Age (years)**	Mean 70	Mean 78	Median 73
**Male (%)**	86	89	91
**Antithrombotic therapy (%)**	30	55	27
**Average ASA score**	NA	ASA 3	NA
**Clinical presentation of haematuria**	All patients admitted to emergency department with macroscopic haematuria	All patients requiring overnight hospitalisation for management of macroscopic haematuria	Patients admitted for gross haematuria with a documented history of pelvic radiation
**Underlying aetiology of bladder cancer (%)**	35	8.3	4.8
**Underlying aetiology of prostate cancer (%)**	1.3	16	86
**Bedside Investigations**	Urine dipstick	NA	NA
**Laboratory Investigations**	Blood tests	NA	NA
**Radiological Investigations (%)**	US 31 CT and/or MRI 69	18 US 12 CT 5.8 US and CT	NA
**Conservative management**	NA	67% catheterised using 3‐way catheter 10% catheterised using 2‐way catheter 62% commenced on continuous bladder irrigation on admission, 85% on Day 1, 12% on Day 2, 1.4% on Day 3 and 1.4% on Day 5 63% manual bladder washout using a bladder syringe	88% continuous bladder irrigation
**Surgical management (%)**	69 surgical intervention	10.0 rigid cystoscopy and bladder washout	80 cystoscopy 58 electrocautery for haemostasis
**LoS (days)**	Mean 7.1	Mean 5.0	Median 9.0
**Transfusion rate (%)**	NA	11	88
**Readmission rate (%)**	NA	30‐day 8.3	90‐day 35
**Mortality rate (%)**	NA	30‐day 5.0	NA

Abbreviations: ASA, American Society of Anesthesiologists Physical Status Classification System; LoS, Length of Stay.

### 3.3 Disease and intervention‐related factors

Underlying aetiologies are presented in Table 1. All three studies reported the incidence of underlying bladder and prostate carcinoma individually. Pooling of incidence in individual studies revealed bladder cancer was present in 17% of patients and, similarly, prostate cancer was present in 17% of patients. A total of 61% of patients underwent radiological investigation. Surgical intervention was carried out in a total of 38% of patients.

Only one article^4^ reported findings from inpatient scans and cystoscopy. Findings in the 36% (43/120) of patients who underwent inpatient scans were: normal imaging (28%), enlarged prostate (19%), bladder wall thickening (14%), renal cysts (14%), calculi (9.3%), bladder mass (7.0%), renal mass (4.7%), hydronephrosis secondary to stone (2.3%) and hydronephrosis secondary to other causes (23%). Findings in the 10% (12/120) of patients who underwent inpatient cystoscopy were: bladder mass (42%), normal (33%), radiation cystitis (25%), vascular prostate (17%), red patch (17%) and other findings (25%) such as trabeculations and diverticulum. None of the studies described the type of care provided, whether it was in a regular ward, a high‐dependency unit or an intensive care unit.

### 3.4 Outcome‐related factors

Two of the three studies reported mean length of stay. Pooling of these two means reveals a mean duration of 5.8 days.^4^, ^10^ Readmission and mortality rates within 30 days from discharge were only reported by one study as 8.3% (10/120) and 5.0% (6/120), respectively.^4^ A 90‐day readmission rate of 35% (9/26) was reported by another study.^11^ This same study reported a median LoS of 9.0 days (IQR 5–17 days). None of the studies reported the number of previous admissions for haematuria, rate of thromboembolic events, complications of procedures performed as part of unscheduled haematuria management or 90‐day mortality.

### 3.5 Management strategies

Sönmez et al.^10^ found 54 patients (69.2%) required surgical management following admission for haematuria. Other 24 (30.8%) patients were managed conservatively with a catheter. Those who were found to have malignancy or underwent a surgical procedure had a longer length of stay and catheterisation duration. No further details are provided in the management strategies for this cohort of patients.

Pavithran et al.^4^ found 93/120 patients (78%) were catheterised, and 74 patients (62%) were commenced on continuous bladder irrigation (CBI), which decreased to 1/74 (1%) on Day 5 of admission. A total of 12/120 patients (10%) did not respond to conservative measures and required cystoscopy and bladder washout to control the bleeding. This included interventions such as as rollerball diathermy (6/12), clots were removed using a bladder syringe (5/12), trans‐urethral resection of bladder tumour (TURBT) (2/12) and biopsies (1/12).

Wallace et al.^11^ found CBI was utilised in 88% of admissions, with 80% requiring cystoscopy and 58% electrocautery. Five different pharmacological agents were used over the study period (alum, aminocaproic acid, carboprost, formalin and pentosan polysulphate sodium) in a total of eight different formulations, including both oral and intravesical modes of delivery. One patient for refractory haematuria required anterior iliac artery embolisation, and another required simple cystectomy with ileal conduit.

### 3.6 Secondary outcomes

A further nine articles did not meet the primary outcome but included relevant secondary outcomes. Six articles provided additional information on the underlying aetiology or incidence of unscheduled haematuria shown in Table 2. A retrospective cohort of 69 patients undergoing flexible cystoscopy reported 38% had findings suspicious for malignancy.^16^ Analysis of emergency department visits for different urological presentations found unscheduled haematuria to be present in 36% of patients presenting with radiation cystitis,^3^ 30% of patients presenting with LUTS^13^ and 1.6% of patients with renal colic.^15^ Post‐procedural readmission for haematuria was prevalent in 3.4% of patients post‐TURBT^12^ and 2.1% of patients post‐TRUS prostate biopsy.^14^

**TABLE 2:** Studies with secondary outcome data reporting underlying aetiology/incidence of emergency haematuria.

	Sarmah et al.^12^	Roghmann et al.^13^	Cheng et al.^14^	Hong et al.^15^	Lee et al.^3^	Ben‐David et al.^16^
**Study type**	Retrospective cohort, multicentre	Retrospective cohort, multicentre	Retrospective cohort, multicentre	Retrospective cohort, multicentre	Retrospective cohort, multicentre	Retrospective cohort
**Year**	2023	2023	2019	2015	2021	2022
**Total number of patients with emergency haematuria**	15	112 985	35	374	10 055	69
**Underlying aetiology/incidence of emergency haematuria**	3.4% of patients undergoing elective TURBT were re‐admitted as emergencies with haematuria within 30 days of TURBT	30% of males treated for LUTS in emergency department had inpatient haematuria	2.1% of men post TRUS prostate biopsy developed gross haematuria requiring emergency attendances 0.82% of men post TRUS prostate biopsy developed gross haematuria requiring emergency attendance with hospitalisation for further management	1.6% of patients presenting to the emergency department with renal colic have chief complaint of haematuria	35.6% of emergency department visits for radiation cystitis among patients with a prostate cancer history had gross haematuria	Flexible cystoscopy findings in patients hospitalised due to gross haematuria 38% with findings suspicious for malignancy: 12% with hyperaemic prostate 19% with oedematous/bullous changes within the bladder 49% with findings not suspicious for malignancy: 27% with normal findings 27% with hyperaemic prostate 62% with oedematous/bullous changes within the bladder 13% with non‐diagnostic exam: 44% with hyperaemic prostate 33% with oedematous/bullous changes within the bladder 100% with severe haematuria, bladder changes limiting diagnostic ability

Abbreviations: LUTS, lower urinary tract symptoms; TURBT, transurethral resection of bladder tumour; TRUS, transrectal ultrasound.

Three articles assessed either the effect of anticoagulation or intervention used in patients admitted with unscheduled haematuria, as shown in Table 3. A single‐centre retrospective cohort study examined the effect of oral anticoagulation on the management of patients with emergency admissions for gross haematuria.^18^ The authors found anticoagulation status to be a significant predictor for intervention. 86% of anticoagulated patients admitted with emergency haematuria required bladder irrigation, compared to 62% of patients not on any anticoagulation. Another single‐centre retrospective study of all patients admitted to the emergency department with visible haematuria reported median LoS according to type of anticoagulation.^2^ Median LoS was reported as 1.0 day for patients on no anticoagulant therapy and 1.0 for patients on direct oral anticoagulants (DOACs). Patients on vitamin K antagonists (VKA) had a median LoS of 3.0 days.

**TABLE 3:** Studies with secondary outcome data reporting the effect of anticoagulation status or intervention.

	Moharamzadeh et al.^17^	Satasviam et al.^18^	Müller et al.^2^
**Study type**	RCT	Retrospective cohort	Retrospective cohort
**Year**	2017	2012	2019
**Total number of patients with emergency haematuria**	50	118	811
**Effect of anticoagulation status or intervention**	Effect of tranexamic acid on patients referred to emergency department with gross haematuria: Tranexamic acid significantly decreased the volume of used serum for bladder irrigation and the microscopic status of urine decreased significantly in terms of the haematuria after 24 hours Rate of packed cell transfusion (64% vs 36%) and drop in haemoglobin levels showed no significant difference between tranexamic acid and placebo groups.	86% of emergency admissions for haematuria where the patient was taking oral anticoagulation required bladder irrigation 62% of emergency admissions for haematuria where the patient was not on any oral anticoagulation required bladder irrigation 7.3% of patients on oral anticoagulation with emergency admission for haematuria required transfusion 4.1% of patients without oral anticoagulation with emergency admission for haematuria required transfusion	Median LoS according to type of anticoagulation: 1.0 day for DOAC 3.0 days for VKA 1.0 day for no anticoagulant therapy

Abbreviations: DOAC, direct oral anticoagulant; VKA, vitamin K antagonist.

Our search identified only one relevant RCT which assessed the effect of tranexamic acid on patients referred to the emergency department with gross haematuria.^17^ Tranexamic acid use for bladder irrigation significantly decreased the volume of required normal saline serum for bladder irrigation, but there was no significant effect on the drop in serum haemoglobin levels or rate of packed cell transfusion.

### 3.7 Risk of bias

**FIGURE 2:**
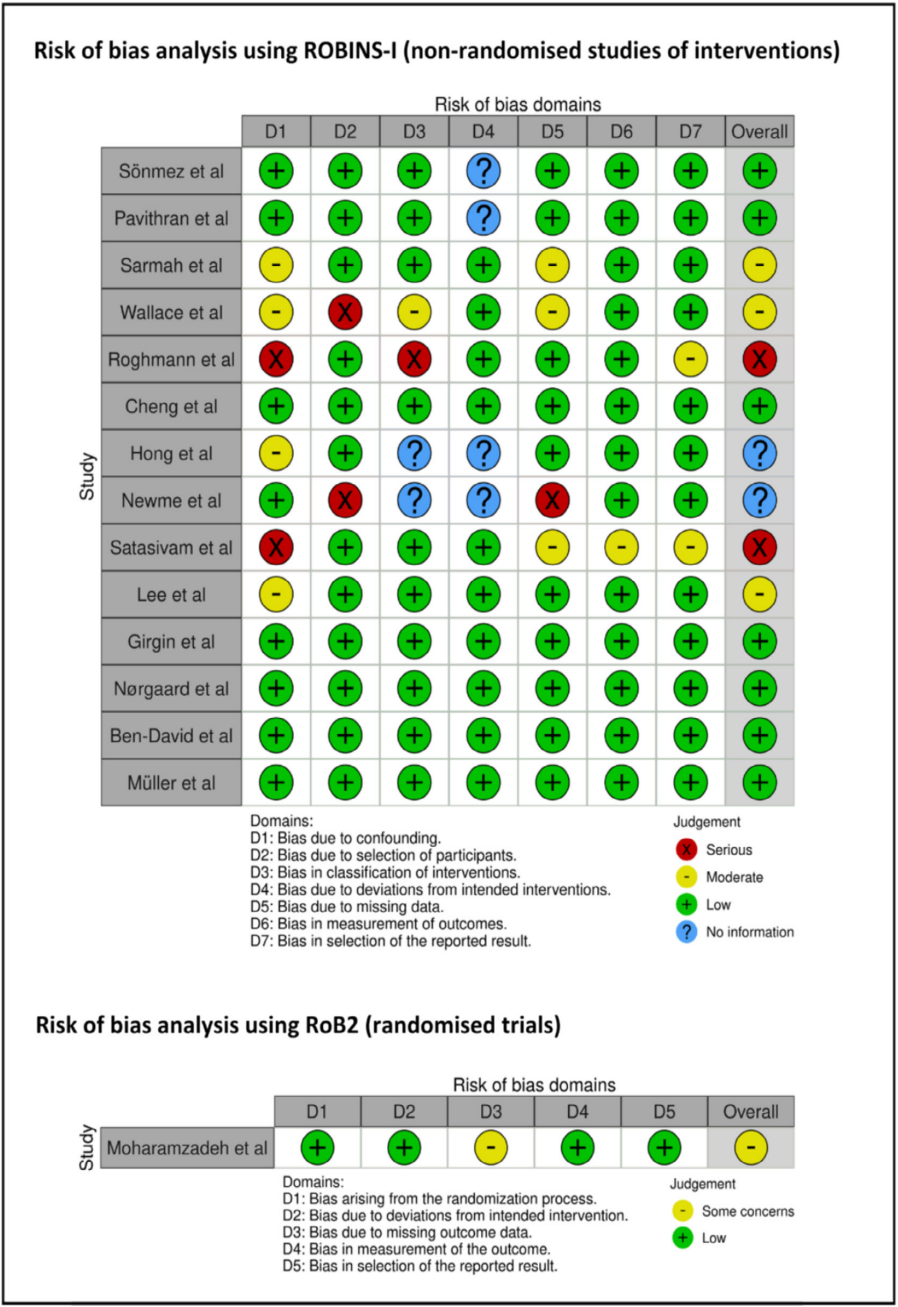
Risk of bias analysis using ROBINS‐I and RoB2 for non‐randomised studies of interventions and randomised trials, respectively.

Visual representations of risk of bias assessment using ROBINS‐I and RoB2 for non‐randomised cohort studies and RCTs, respectively, are shown in Figure 2. The overall quality of studies was moderate, with seven having low risk of bias.

## 4 DISCUSSION

To the authors' knowledge, this is the first systematic review investigating LoS and the current management strategies in patients admitted with unscheduled emergency haematuria. This systematic review found limited relevant research with only three studies addressing our primary outcome, highlighting a lack of reported data for this common urological emergency. There was variation in the types of outcomes reported for this cohort of patients; however, the overall mean LoS after pooling reported means was 5.8 days. The included study populations comprised an older and predominantly male patient population with a pooled mean age of 75 years and 88% male with 17% of patients having underlying bladder cancer and similarly 17% of patients having underlying prostate cancer. This reflects the demographics of real‐world disease population. A project in collaboration between three areas in the North of England and Better Care Fund programme reported that 36% of patients delayed in hospitals are due to lack of a named responsible consultant or team for patients and clear communication of patient's disease outcome.^19^ This demonstrates the need for standardised guidelines for managing patients admitted with haematuria.

The prevalence of antithrombotic therapy use in these patients is high, with a pooled prevalence of 45.0%. A retrospective cohort study of 811 patients found those taking VKAs had more hospitalisation episodes and longer LoS than those on DOACs and no difference between patients on DOAC versus no anticoagulants.^2^ Further research is needed to establish whether there may be a benefit in switching patients with unscheduled haematuria from VKA to DOAC therapy.

Little evidence exists on the timing and use of radiological investigations. A pooled 61% of patients underwent radiological investigations as an inpatient, which may be a missed opportunity for some patients to receive an expedited diagnosis or treatment during the inpatient stay. In the absence of high‐quality evidence demonstrating benefit, clinicians in certain countries may prefer to rely on fast‐track outpatient investigations. It remains unclear if this is beneficial in all patients, or whether certain cohorts of patients may even have an expedited discharge due to findings on radiology. In addition to this, some patients may experience delays in oncological diagnosis such as muscle‐invasive bladder cancer. In the IDENTIFY study, focussing specifically on patients admitted as an emergency with visible haematuria, 32% of patients had an underlying malignant diagnosis.^8^

Furthermore, evidence is required to guide the timing and threshold for intervention. With a pooled intervention rate of 38%, conservative management is futile for a significant proportion of patients. This impacts on resource use and patient experience, particularly in those with an already long inpatient stay before the decision is made to take to the theatre. However, there is no evidence to guide patient selection and predict who will fail conservative management. Data from large‐scale observational studies may aid the development of a patient selection process for appropriate inpatient investigations and timely intervention.

Oral and intravesical therapies were trialled in those with radiation‐induced haemorrhagic cystitis and found 90‐day readmission rates of 33% for intravesical therapy (3/9) and 50% for oral therapy (1/2).^11^ Further research is required in the use of oral and intravesical therapies and clear evidence for treatment sequencing.^11^ Surgical interventions included electrocautery, biopsies, clot removal and TURBT. It was not made clear if the TURBTs were for palliative or curative intent. There was no mention of the use of androgen deprivation therapy or the use of 5‐alpha reductase for the management of prostate cancer and BPH, respectively. This highlights the need for high‐quality prospective data to understand management strategies and their outcomes.

The limitations of this systematic review relate to the limitations of the primary included studies, all of which were retrospective cohort studies with risk of bias assessment demonstrating a low to moderate risk. Additionally, the heterogeneity in reported outcomes limited the ability to pool data to answer the research question underpinning this systematic review and meant meta‐analysis was not possible.

## 5 CONCLUSIONS

Unscheduled haematuria admissions represent a significant challenge in urological practice, with a notable gap in evidence‐based protocols to guide management. Despite its common occurrence, particularly among older male patients with comorbidities such as antithrombotic therapy use and underlying urological malignancies, there is a striking lack of relevant research. This is reflected by only three studies meeting the eligibility criteria in this first systematic review in this area. The condition is associated with high cancer diagnosis rates and prolonged hospital stays, with a pooled mean length of stay of 5.8 days.

To address this issue, there is a critical need for multicentre prospective studies to develop standardised protocols. These efforts could significantly improve patient outcomes by addressing the current shortcomings in the management of this prevalent urological emergency.

## AUTHOR CONTRIBUTIONS

*Study design*: Nikki Kerdegari, Raghav Varma, Simona Ippoliti, Cameron Alexander, Kevin Byrnes, Nikita Bhatt and Veeru Kasivisvanathan. *Data analysis*: Nikki Kerdegari, Raghav Varma and Simona Ippoliti. *Article draft*: Nikki Kerdegari, Raghav Varma, Simona Ippoliti, Cameron Alexander, Kevin Gallagher and Sinan Khadhouri. *Critical revision*: Arjun Nathan, Kevin Byrnes, Nikita Bhatt and Veeru Kasivisvanathan. *Publication approval*: All authors.

## CONFLICT OF INTEREST STATEMENT

The authors declare no conflicts of interest.

## Supporting information


**Data S1.** Supporting information.
