# Supplementary material for: A systematic review of outcomes associated with patients admitted to hospital with emergency haematuria
Source: BJUI Compass. 2025 Feb 17;6(2):e497. doi: 10.1002/bco2.497 (PMC11832295; doi:10.1002/bco2.497)
Supplement: Supplementary file 1 — Data S1. Supporting information. [file BCO2-6-e497-s001.docx]

**Appendix 1: Search strategy for systematic review**

**Database: Embase <1974 to 2023 October 06>**; **Ovid MEDLINE(R) ALL <1974 to 2023 October 06>**

1. Inpatient.mp. or exp hospital patient/ (426598)
2. exp emergency/ (99276)
3. unscheduled.mp. (13291)
4. unplanned.mp. (50300)
5. exp hospital admission/ or admission.mp. (862615)
6. haematuria.mp. or exp hematuria/ or “clot retention”.mp. (78082)
7. exp management/ (1529359)
8. treatment.mp. (14442252)
9. exp therapy/ (15610014)
10. intervention.mp. (2122680)
11. exp surgery/ (5874540)
12. 1 or 2 or 3 or 4 or 5 (1342080)
13. 6 and 12 (4852)
14. 7 or 8 or 9 or 10 or 11 (27212004)
15. 13 and 14 (4406)
16. conference abstract.pt. or Congresses as Topic/ or Conference Review.pt. or "Journal: Conference Abstract".pt. (5053498)
17. 15 not 16 (2805)
18. Limit 16 to English language (2574)
19. Remove duplicates from 18 (2426)

**Updated Search Strategy**

**Database: Embase <1974 to 2023 October 06>**

1. Inpatient.mp. or exp hospital patient/ (320267)
2. exp emergency/ (55983)
3. unscheduled.mp. (7938)
4. unplanned.mp. (31409)
5. exp hospital admission/ or admission.mp. (601604)
6. haematuria.mp. or exp hematuria/ or “clot retention”.mp. (61866)
7. exp management/ (1529359)
8. treatment.mp. (8500450)
9. exp therapy/ (10356311)
10. intervention.mp. (1287339)
11. exp surgery/ (5833935)
12. 1 or 2 or 3 or 4 or 5 (932934)
13. 6 and 12 (4473)
14. 7 or 8 or 9 or 10 or 11 (17425501)
15. 13 and 14 (4177)
16. conference abstract.pt. or Congresses as Topic/ or Conference Review.pt. or "Journal: Conference Abstract".pt. (5024775)
17. 15 not 16 (2576)
18. Limit 16 to English language (2398)

**Updated Search Strategy**

**Database: Ovid MEDLINE(R) ALL <1974 to 2023 October 06>**

1. Inpatient.mp. or exp hospital patient/ (106331)
2. exp emergency/ (43293)
3. unscheduled.mp. (5353)
4. unplanned.mp. (18891)
5. exp hospital admission/ or admission.mp. (261011)
6. haematuria.mp. or exp hematuria/ or “clot retention”.mp. (16216)
7. exp management/ (0)
8. treatment.mp. (5941802)
9. exp therapy/ (5253703)
10. intervention.mp. (835341)
11. exp surgery/ (40605)
12. 1 or 2 or 3 or 4 or 5 (409146)
13. 6 and 12 (379)
14. 7 or 8 or 9 or 10 or 11 (9786503)
15. 13 and 14 (229)
16. conference abstract.pt. or Congresses as Topic/ or Conference Review.pt. or "Journal: Conference Abstract".pt. (28723)
17. 15 not 16 (229)
18. Limit 16 to English language (176)

**Updated Search Strategy**

**Database: Web of Science**

**Search Performed 08/10/2023**

#1 ALL=((Inpatient OR "hospital patient" OR emergency OR unscheduled OR unplanned OR "hospital admission" or admission)) (1035558)

#2 ALL=((haematuria OR hematuria OR “clot retention”)) (19617)

#3 ALL=((management OR treatment OR therapy OR intervention OR surgery)) (15671471)

#1 AND #2 AND #3 (1051)
